# Acetic acid is a superior acidifier for sub-nanogram and single cell proteomic studies

**DOI:** 10.1101/2023.08.01.551522

**Published:** 2023-08-02

**Authors:** Benjamin C. Orsburn

**Affiliations:** The Department of Pharmacology and Molecular Sciences, Single Cell Proteomics Center, The Johns Hopkins University School of Medicine, Baltimore, MD, USA, 21205

## Abstract

A recent study demonstrated a substantial signal increase when employing a 0.5% acetic acid buffer additive instead of the traditional 0.1% formic acid used in shotgun proteomics. In this study I compare these two buffers for a dilution series of tryptic digests down to 20 picograms peptide on column on a TIMSTOF single cell proteome (SCP) system. I observe a comparable relative level of signal increase as previously reported, which translates to improvements in proteome coverage at every peptide load assessed. The relative increase in peptide identifications is more apparent at lower concentrations with a striking 1.8-fold more peptides identified at 20 pg peptide load, resulting in over 2,000 protein groups identified in 30 minutes on this system. These results translate well to isolated single human cancer cells allowing over 1,000 protein groups to be identified in single human cells processed using a simple one step method in standard 96-well plates.

All vendor raw and processed data has been made publicly available at www.massive.ucsd.edu and can be accessed as MSV000092563.

## Graphical Abstract

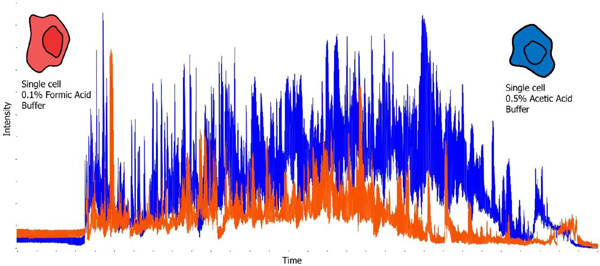

## Introduction

Single cell proteomics by LCMS is a growing field of research driven by rapid innovation in sample preparation^1,2^, chromatography^3^, mass spectrometry methods^4–6^, instrumentation^7^ and new data analysis solutions.^8–10^ Despite these advances we continue to deal with a finite amount of material in an extremely complex form and any advance to increase peptide detection efficiency should be explored. A recent work by Battellino *et al*., described a simple solution to increase peptide signal in LCMS experiments by simply substituting the 0.1% formic acid used in nearly every proteomics workflow with 0.5% acetic acid.^11^ While this group noted some decreases in chromatographic resolution, they observed a nearly 2.5-fold increase in peptide signal. While this may be of somewhat limited use in most shotgun proteomics where we are not typically sample limited this could be a remarkable improvement if leveraged in single cell shotgun proteomics.

## Methods

### Preparation of K562 dilution series

Promega human cancer cell line tryptic digest (V7461) was serial diluted using a solution containing 0.1% formic acid in LCMS grade water (Pierce 85170) and 0.1% DDM (Fisher 89902). A total of 10 microliters of each dilution, 10 pg/µL, 100 pg/µL, and 1000 pg/µL, were loaded into 16 wells each of a 96 well plate. The plate was tightly sealed with plate sealing adhesive tape (Fisher, 60180-M143) and centrifuged prior to loading on the autosampler.

### LCMS instrument parameters

Prior to LCMS analysis the TIMSTOF SCP was freshly tuned using the OTOF Control engineering software with an infusion of 3 µL/min of low-concentration tuning mix (Agilent, G1969-85000). Autotuning was performed for sensitivity and resolution tuning was manually checked, but tuning values obtained during the resolution tune were not accepted as they led to a noticeable decrease in intensity in the 622 and 922 calibrant ions (data not shown).

An EasyNLC 1200 system (Proxeon) coupled to a TIMSTOF SCP (Bruker Daltronic) was used for all analyses. Peptides were separated using a gradient with a constant flow of 300 nL/min using an IonOpticks 15 cm x 75 µm C-18 column with 1.5 µm particle size (Ion Opticks “Ultimate”) with integrated CaptiveSpray emitter held at 1500V. For standard injections, 2 µL of each well was loaded with a partial loop injection of 7 µL at 900 bar. For single cell injections 4 µL was picked up with a partial loop injection volume of 10 µL to counter for peptide diffusion within the sample loop. Buffer A consisted of LCMS grade water and either 0.1% formic acid or 0.5% acetic acid as appropriate. Buffer B consisted of 80% LCMS grade acetonitrile in water with the appropriate acidifier for each experiment. The 30 minute gradient used in all experiments began at 8% buffer B and ramped to 35% B by 22 minutes. The gradient then increased to 100% B by 26 minutes where it held for 2 minutes before returning to baseline conditions. The column was equilibrated in 12 microliters of baseline conditions prior to each injection.

The TIMSTOF SCP system was operated in diaPASEF mode using a method with 50 Da isolation windows provided by Dr. Michael Krawitzky of Bruker Daltronic during instrument training. In this method, the MS1 scan is obtained from 100–1700 m/z with a 1/k0 window of 0.6 – 1.4. Ions that enter the mass analyzer in these systems are restricted by a user defined polygon that imparts dramatic alterations on ion signal obtained and must be carefully optimized for each series of experiments. In this case, the polygon began at 300 m/z and attempted to not acquire any ions in the MS1 cloud below 800 m/z. The method utilizes 6 cycles with 3 isolation windows per cycle resulting in a total time of 1.2 seconds when a 166 ms ramp time is used. The “high sensitivity” mode was enabled for all samples. For the 100 ms experiments described the only alteration in the method was that the ramp time was decreased from 166ms to 100ms to allow a shorter cycle time of 0.72 seconds.

### Isolation and preparation of SW620 and PANC0203 cancer cells

Cancer cell lines were obtained from ATCC and grown in the appropriate culture media described by the vendor. For PANC 0203 this was RPMI 1640 (ATCC 30-2001) supplemented with 15% fetal bovine serum (ATCC 30-2020) and 10 units of human insulin (Fisher). SW620 cells were grown in Leibovitz’s L-15 Medium (ATCC 30-2008) supplemented with 10% fetal bovine serum (ATCC 30-2020). Culture media was supplemented with 10 mg/mL Penn Strep antibiotic solution (ATCC 30-2300). All cell lines were passaged a minimum of 3 times prior to single cell isolation. Cells were harvested first by vacuum aspiration of the cell culture media. The adherent cells were briefly rinsed in 3 mL of 0.05% Trypsin plus EDTA solution (ATCC 30-2001). This solution was rapidly aspirated off and replaced with 3 mL of the same solution. The cells were examined by light field microscopy and incubated at 37°C with multiple examinations until the adherent cells had lifted off the plate surface. The active trypsin was then quenched by the addition of 7 mL of the original culture media. The 10 mL solution was transferred to sterile 15 mL Falcon tubes (Fisher) and centrifuged at 300 *x g* for 3 minutes to pellet the cells. The supernatant was gently aspirated off and the cells were resuspended in PBS solution without calcium or magnesium with 0.1% BSA (both, Fisher Scientific) at 1 million cells per mL as estimated by bright field microscopy. Cells for single cell aliquoting were gently dissociated from clumps by slowly pipetting a solution of approximately 1 million cells through a Falcon cell strainer (Fisher, 353420) and the cells were placed on wet ice and immediately transported to the JHU Public Health sorting core. Non-viable cells were labeled with a propidium iodide solution provided by the core facility and briefly vortexed prior to cell isolation and aliquoting.

Single cells were aliquoted using an analog MoFlo sorter into cold 96 well plates containing 2 microliters of LCMS grade acetonitrile. At the completion of each plate aliquoting they were immediately sealed and placed in an insulated box of dry ice with the wells pressed into the material to ensure rapid cooling. The frozen single cells were transported back to −80C storage (PANC 0203) or processed immediately (SW620). For processing, acetonitrile was driven off by heating the cells for 90 seconds on a hotplate at 95°C. Dried cell lysate was digested using a solution of 5 nanogram/microliter LCMS grade trypsin (Pierce) in 0.1% n-Dodecyl-beta-Maltoside Detergent (DDM, Thermo Fisher, 89902) and 50mM TEAB. Two microliters of trypsin solution were used for each cell prior to the plate being tightly sealed with adhesive plate tape (Fisher, 60180-M143) and room temperature overnight digestion. Following digestion, the peptide digest was briefly centrifuged to condense evaporation and the plates were completely dried with vacuum centrifugation. The peptides were resuspended in 3.5 µL of 0.1% formic acid, vortexed and centrifuged prior to loading on the autosampler.

### LCMS instrument parameters

Prior to LCMS analysis the TIMSTOF SCP was freshly tuned using the OTOF Control engineering software with an infusion of 3 µL/min of low-concentration tuning mix (Agilent, G1969-85000). Autotuning was performed for sensitivity and resolution tuning was manually checked, but tuning values obtained during the resolution tune were not accepted as they led to a noticeable decrease in intensity in the 622 and 922 calibrant ions prior to sensitivity tuning alone (data not shown).

An EasyNLC 1200 system (Proxeon) coupled to a TIMSTOF SCP (Bruker Daltronic) was used for all analyses. Peptides were separated using a gradient with a constant flow of 300 nL/min using an IonOpticks 15 cm x 75 µm C-18 column with 1.5 µm particle size (Ion Opticks “Ultimate”) with integrated CaptiveSpray emitter. For standard injections, 2 µL of each well was loaded with a partial loop injection of 7 µL at 900 bar. For single cell injections 4 µL was picked up with a partial loop injection volume of 10 µL to counter for peptide diffusion within the sample loop. Buffer A consisted of 100% aqueous and either 0.1% formic acid or 0.5% acetic acid. Buffer B consisted of the same with 80% LCMS grade acetonitrile in water with the appropriate acidifier for each experiment. The gradient in all experiments began at 8% buffer B and ramped to 35% B by 22 minutes. The gradient rapidly increased to 100% B by 26 minutes where it held for 2 minutes before returning to baseline conditions. The column was equilibrated in 12 microliters of baseline conditions prior to each injection.

The TIMSTOF SCP system was operated in diaPASEF mode using a method with 50 Da isolation windows provided by Dr. Michael Krawitzky of Bruker Daltronic during instrument training. In this method, the MS1 scan is obtained from 100–1700 m/z with a 1/k0 window of 0.6 – 1.4. The method utilizes a 6 cycle method with 3 isolation windows per cycle resulting in a total cycle time of 1.2 seconds when a 166 ms ramp time is used. The “high sensitivity” mode was enabled for all samples. For the 100 ms experiments described the only alteration in the method was that the ramp time was decreased from 166ms to 100ms to allow a shorter cycle time of 0.72 seconds.

Between experiment sets, buffers were switched, and 5 purge cycles were ran on the instrument followed by a 10 microliter precolumn and 12 microliter analytical column equilibration at 900 bar to prepare the instrument for replicate samples ran with the next buffer combination.

### Data Analysis

SpectroNaut 18 (Biognosys) was used for all data analysis using the directDIA+ workflow and a human Uniprot library utilizing all default parameters for data calibration and analysis. All files were first converted to HTRMS using the appropriately named “HTRMS Converter” software from Biognosys prior to analysis. Output data was visualized in GraphPad Prizm 10.0.1.

## Results

### Acetic acid improves proteomic coverage on sub-nanogram injections of peptides

**Figure 1.**
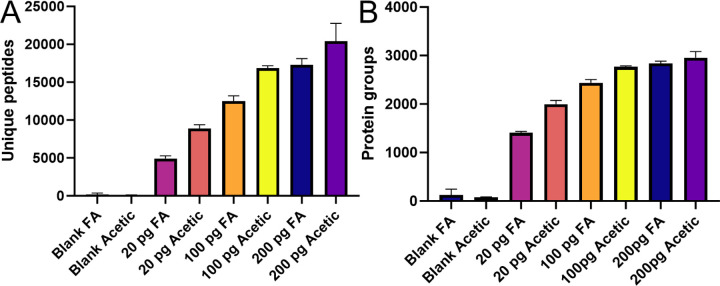
A summary of identifications obtained with sub-nanogram injections of a commercial tryptic digest standard. **A.** Unique peptides identified at 20, 100 and 200 picogram of peptide digest with 0.1% formic acid (FA) or 0.5% acetic acid (Acetic) in the running buffers. **B.** The same analysis with protein groups identified.

In agreement with the results reported by Battellino *et. al*.,^11^ I observed an increase in detected precursors, peptides, proteins and protein groups in every injection of a K562 standard below 400 picogram total load on column. At 400 pg or above, precursors and peptide groups are higher, but this does not translate to a significant increase in protein groups detected. As peptide concentration on column decreased the relative number of identifications gained by acetic acid buffer increased. Remarkably, at 20 picogram K562 digest on column, peptide and protein identifications nearly double leading to the striking observation of 2,000 proteins identified at this concentration (Figure 1, Supplemental File 1).

### Increased relative signal from acetic acid buffer can be used to obtain more scans across each peak.

**Figure 2.**
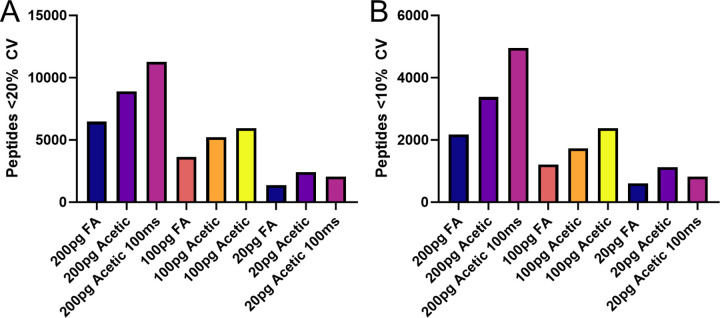
A summary of the number of peptides quantified with %CVs below certain cutoffs. **A.** Peptides quantified at less than 20% CV when injecting the same concentration of peptides with formic acid buffer (FA), acetic acid buffer with default ramp time (Acetic) or the same with a reduced ramp time of 100ms. **B.** The same samples with numbers of peptides quantified at less than 10% CV shown.

While obtaining higher numbers of peptides is one use of the increased relative signal provided by acetic buffer, it could also be used in other ways such as reducing relative ion accumulation time and allowing more scans across each peak. The default ramp time on the TIMSTOF SCP system and vendor provided methods is 166 ms, resulting in a cycle time of 1.2 seconds. By decreasing the ramp time to 100ms the instrument software estimated a 0.7 second cycle time, or 1.7 times more scans across each peak. In general, more measurements allow improved relative quantification, which is typically assessed in shotgun proteomics by calculating the coefficient of variation (%CV).^12^ As shown in Figure 2, the number of peptides with lower CVs increased in both the 200 picogram and 100 picogram replicates. At 20 picograms on column, however, the quantitative accuracy decreased, suggesting that this higher ion accumulation time is required to obtain quantitative data at peptide levels this low.

### Acetic acid dramatically increases proteomic coverage in isolated single human cells

**Figure 3.**
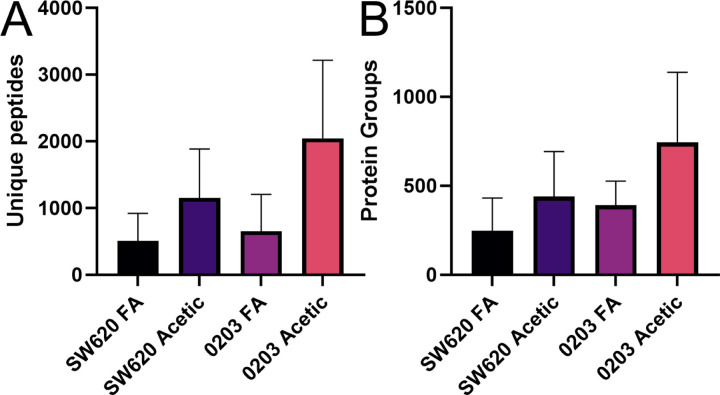
Peptides and proteins obtained from single cells prepared in 96-well plates using a single step digestion method. **A.** Average and mean unique peptides from SW620 and PANC 0203 cells analyzed using the same method with 0.1% formic acid (FA) or 0.5% acetic acid (Acetic) buffer additive. **B.** The protein groups from this same set of experiments.

In two cancer cell lines analyzed, the results closely mimic those of the K562 peptide dilution series, with large improvements in precursors, peptides and proteins identified in each single cell obtained simply by swapping running buffers. Results were more impressive in the PANC 0203 cell line than SW620, for reasons that are beyond the scope of this technical note (Figure 3). Remarkably, using this relatively simple and fast preparation and analysis method, multiple cells were observed with more than 1,000 protein groups identified (Supplemental File 1), a number that is generally obtained only with careful monitoring of sample loss with picoliter robotics^1,5^ or other advanced systems.^13^

## Conclusions

Adulterating running buffers to increase peptide signal and identification rates is not a new concept. The addition of 5% DMSO has long been noted as an additive that can lead to impressive increases in peptide and protein coverage relative to 0.1% formic acid alone.^14^ As noted in Battellino *et. al.,* however, less than 2% of all proteomics studies in the literature have employed DMSO.^11^ While I have personally used DMSO in proteomics experiments, I have experienced severe contamination issues after long term use of the that required extensive cleaning of internal instrument components (data not shown). Acetic acid appears to provide comparable increases in peptide signal and has been employed in LCMS systems used for the analysis of extractables and leachables and pesticides for decades.^15,16^ It seems highly unlikely, for this reason, to be detrimental to long term instrument performance. More careful sample preparation methods resulting in higher levels of starting peptide materials from each single cell will undoubtedly improve the gap in coverage between the peptide standards and single cells analyzed in this study.

## Supplementary Material

Supplement 1
