# Supplementary material for: Acetic acid is a superior ion pairing modifier for sub-nanogram and single cell proteomics
Source: bioRxiv. 2024 Jun 10:2023.08.01.551522. Originally published 2023 Aug 2. Preprint. [Version 2] doi: 10.1101/2023.08.01.551522 (PMC10418182; doi:10.1101/2023.08.01.551522)
Supplement: 1 [file NIHPP2023.08.01.551522V2-supplement-1.pdf]

bioRxiv preprint doi: https://doi.org/10.1101/2023.08.01.551522; this version posted June 10, 2024. The copyright holder for this preprint (which was not certified by peer review) is the author/funder, who has granted bioRxiv a license to display the preprint in perpetuity. It is made available under aCC-BY 4.0 International license.

Supplemental Figure 1

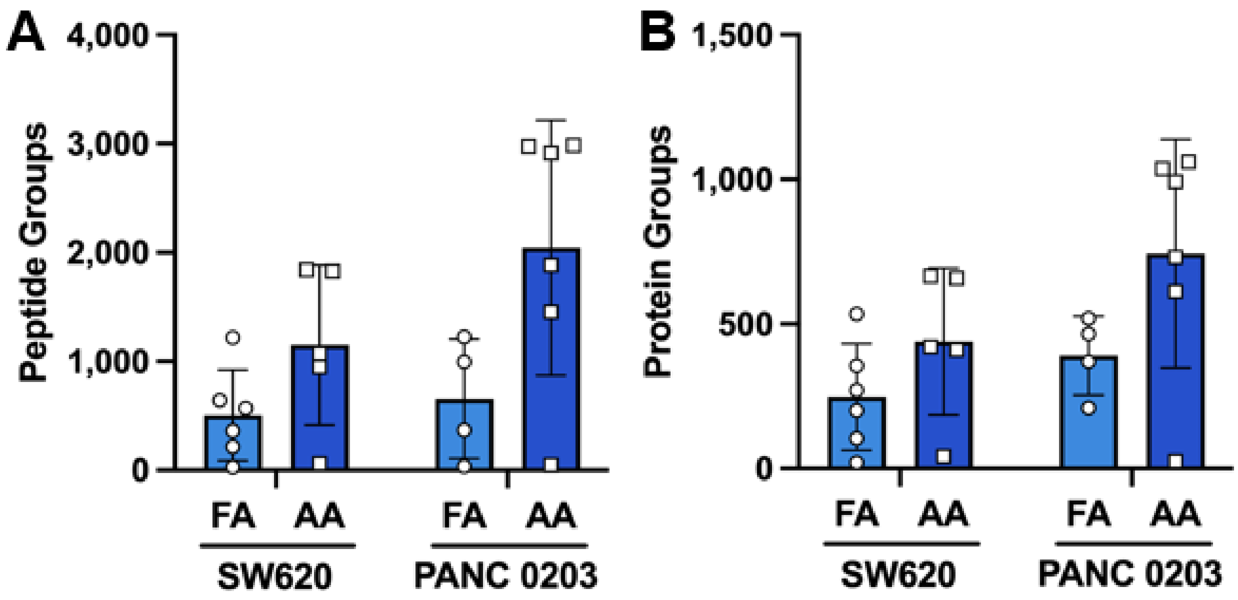

Supplemental Figure 1. Peptides and proteins obtained from single cells. (A) Peptide and **(B)** protein groups identified in SW620 and PANC 0203 cells analyzed using a single step digestion method and run using 0.1% formic acid (FA) or 0.5% acetic acid (AA) buffer additives. Error bars represent mean and standard deviation. Statistical analyses were performed using a two-tailed t-test and FA vs AA comparisons resulted in no significant differences.
